# Supplementary material for: Tunable Fluorescence via Self-Assembled Switching of AIE-Active Micelle-like Nanoaggregates
Source: Int J Mol Sci. 2023 Jun 9;24(12):9941. doi: 10.3390/ijms24129941 (PMC10298664; doi:10.3390/ijms24129941)
Supplement: Supplementary file 1 [file ijms-24-09941-s001.zip › ijms-2421311-supplementary.pdf]

International Journal of
Molecular Sciences

MDPI

Tunable Fluorescence via Self-Assembled Switching of AIE-Active Micelle-like Nanoaggregates

Amal Farghal Noreldein Elsyed [1], Gah-Lai Wong [1], Mohamed Ameen [1], Min-Wei Wu [1] and Cheng-Chung Chang [1,2,*]

[1] Graduate Institute of Biomedical Engineering, National Chung Hsing University, 145 Xingda Road, Taichung 402, Taiwan
[2] Intelligent Minimally-Invasive Device Center, National Chung Hsing University, Taichung 402, Taiwan
* Correspondence: ccchang555@dragon.nchu.edu.tw; Tel.: +886-4-22840734 (ext. 640)

METHODS

1-bromo-4-ethoxybenzene(2a): Add 4-bromophenol (0.52 g, 3.0 mmol) and K_2CO_3 (1.2 g, 9.0 mmol) into a 100 mL double-necked round bottom flask and use 20 mL of acetone to dissolve the compound. Then slowly add 1-iodoethane (360 μL, 4.5 mmol) and react under reflux for 12 hours. The solvent was removed under reduced pressure. Extracted twice with CH_2Cl_2/H_2O, and the organic layer was dried over anhydrous magnesium sulfate ($MgSO_4$). Purify via silica column chromatography (the eluent was n-hexane) to obtain a transparent liquid with a yield of 80 %. ^{1}H NMR (400 Hz, $CDCl_3$): δ = 7.36 (d, J = 9.2 Hz, 2H), 6.77 (d, J = 9.2 Hz, 2H), 3.99 (q, J = 7.0 Hz, 2H), 1.40 (t, J = 7.0 Hz, 3H) ppm.

1-bromo-4-butoxybenzene(4a): Add 4-bromophenol (0.52 g, 3.0 mmol) and K_2CO_3 (1.2 g, 9.0 mmol) into a 100 mL double-necked round bottom flask and use 20 mL of acetone to dissolve the compound. Then slowly add 1-iodobutane (340 μL, 4.5 mmol) and react under reflux for 12 hours. The solvent was removed under reduced pressure. Extracted twice with CH_2Cl_2/H_2O, and the organic layer was dried over anhydrous magnesium sulfate ($MgSO_4$). Purify via silica column chromatography (the eluent was n-hexane) to obtain a transparent liquid with a yield of 80 %. ^{1}H NMR (400 Hz, $CDCl_3$): δ = 7.36 (d, J = 9.2 Hz, 2H), 6.77 (d, J = 9.2 Hz, 2H), 3.92 (t, J = 7.0Hz, 2H), 1.75 (m, 2H), 1.48(m, 2H), 0.97 (t, J = 7.0Hz, 3H) ppm.

1-bromo-4-hexyloxybenzene(6a): Add 4-bromophenol (0.52 g, 3.0 mmol) and K_2CO_3 (1.2 g, 9.0 mmol) into a 100 mL double-necked round bottom flask and use 20 mL of acetone to dissolve the compound. Then slowly add 1-bromohexane (630 μL, 4.5 mmol) and react under reflux for 12 hours. The solvent was removed under reduced pressure. Extracted twice with CH_2Cl_2/H_2O, and the organic layer was dried over anhydrous magnesium sulfate ($MgSO_4$). Purify via silica column chromatography (the eluent was n-hexane) to obtain a transparent liquid with a yield of 80 %. ^{1}H NMR (400 Hz, $CDCl_3$): δ= 7.36 (d, J = 9.2 Hz, 2H), 6.77 (d, J = 9.2 Hz, 2H), 4.06(t, J = 7.0Hz, 2H),1.85 (m, 2H), 1.53 (m, 2H), 1.38(m, 4H), 0.93 (t, J = 7.0 Hz, 3H) ppm.

1-bromo-4-dodecyloxybenzene(12a): Add 4-bromophenol (0.52 g, 3.0 mmol) and K_2CO_3 (1.2 g, 9.0 mmol) into a 100 mL double-necked round bottom flask and use 20 mL of acetone to dissolve the compound. Then slowly add 1-bromododecane (1080 μL, 4.5 mmol) and react under reflux for 12 hours. The solvent was removed under reduced pressure. Extracted twice with CH_2Cl_2/H_2O, and the organic layer was dried over anhydrous magnesium sulfate ($MgSO_4$). Purify via silica column chromatography (the eluent was n-hexane) to obtain a transparent liquid with a yield of 80 %. ^{1}HNMR(400 Hz, $CDCl_3$): ： δ = 7.36 (d, J = 9.2 Hz, 2H), 6.77 (d, J = 9.2 Hz, 2H), 3.91 (t, J = 7.0 Hz, 2H), 1.76 (m, 2H), 1.44(m, 2H), 1.26(m, 16H), 0.88(t, J = 7.0 Hz, 3H) ppm

2-(4-methoxyphenyl)-4,4,5,5-tetramethyl-1,3,2-dioxaborolane(1b): Purge the high-pressure bottle by nitrogen for about 1 minute, then put $Pd(PPh_3)_2Cl_2$ (30 mg) in the high-pressure bottle, and then mix 4-bromoanisole (0.47 g, 2.5 mmol) and the mixed solvent Et_3N/1,4-dioxane = 1/3 (v/v) of 12 mL into it and stir evenly with a magnet. Finally, add pinacoborane (630 μL, 3.75 mmol) and bubble with nitrogen for about 2 minutes. After refluxing for 1 day, the mixture was cooled down to room temperature. Extracted twice with CH_2Cl_2/H_2O, and the organic layer was dried over anhydrous magnesium sulfate ($MgSO_4$). Purify via silica column chromatography (the eluent was n-hexane) to obtain transparent liquid. The yield is about 70%. ^{1}HNMR (400 Hz, $CDCl_3$): δ = 7.75 (d, J = 9.2 Hz, 2H), 6.89 (d, J = 9.2 Hz, 2H), 3.82 (s, 3H), 1.33(s, 12H) ppm.

2-(4-ethoxyphenyl)-4,4,5,5-tetramethyl-1,3,2-dioxaborolane(2b): Purge the high-pressure bottle by nitrogen for about 1 minute, then put $Pd(PPh_3)_2Cl_2$ (30 mg) in the high-pressure bottle, and then mix **2a** (0.50 g, 2.5 mmol) and the mixed solvent Et_3N/1,4-dioxane = 1/3 (v/v) of 12 mL into it and stir evenly with a magnet. Finally, add pinacoborane (630 μL, 3.75 mmol) and bubble with nitrogen for about 2 minutes. After refluxing for 1 day, the mixture was cooled down to room temperature. Extracted twice with CH_2Cl_2/H_2O, and the organic layer was dried over anhydrous magnesium sulfate ($MgSO_4$). Purify via silica column chromatography (the eluent was n-hexane) to obtain transparent liquid. The

yield is about 70%. ^{1}HNMR (400 Hz, $CDCl_3$): δ= 7.74 (d, J = 9.2 Hz, 2H), 6.88 (d, J = 9.2 Hz, 2H), 4.06 (q, J = 7.0 Hz, 2H),1.41 (t, J = 7.0 Hz, 3H), 1.35(s,12H) ppm.

2-(4-butoxyphenyl)-4,4,5,5-tetramethyl-1,3,2-dioxaborolane(4b): Purge the high-pressure bottle by nitrogen for about 1 minute, then put $Pd(PPh_3)_2Cl_2$ (30 mg) in the high-pressure bottle, and then mix **4a** (0.50 g, 2.5 mmol) and the mixed solvent Et_3N/1,4-dioxane = 1/3 (v/v) of 12 mL into it and stir evenly with a magnet. Finally, add pinacoborane (630 μL, 3.75 mmol) and bubble with nitrogen for about 2 minutes. After refluxing for 1 day, the mixture was cooled down to room temperature. Extracted twice with CH_2Cl_2/H_2O, and the organic layer was dried over anhydrous magnesium sulfate ($MgSO_4$). Purify via silica column chromatography (the eluent was n-hexane) to obtain transparent liquid. The yield is about 70%. ^{1}HNMR (400 Hz, $CDCl_3$): δ= 7.74 (d, J = 9.2 Hz, 2H), 6.88 (d, J = 9.2 Hz, 2H), 3.98 (t, J = 7.0 Hz, 2H), 1.75 (m, 2H), 1.48(m, 2H), 0.97 (t, J = 7.0 Hz, 3H) ,1.33 (s, 12H) ppm.

2-(4-(hexyloxy)phenyl)-4,4,5,5-tetramethyl-1,3,2-dioxaborolane(6b): Purge the high-pressure bottle by nitrogen for about 1 minute, then put $Pd(PPh_3)_2Cl_2$ (30 mg) in the high-pressure bottle, and then mix **6a** (0.50 g, 2.5 mmol) and the mixed solvent Et_3N/1,4-dioxane = 1/3 (v/v) of 12 mL into it and stir evenly with a magnet. Finally, add pinacoborane (630 μL, 3.75 mmol) and bubble with nitrogen for about 2 minutes. After refluxing for 1 day, the mixture was cooled down to room temperature. Extracted twice with CH_2Cl_2/H_2O, and the organic layer was dried over anhydrous magnesium sulfate ($MgSO_4$). Purify via silica column chromatography (the eluent was n-hexane) to obtain transparent liquid. The yield is about 70%. ^{1}HNMR (400 Hz, $CDCl_3$): δ= 7.74 (d, J = 9.2 Hz, 2H), 6.89 (d, J = 9.2 Hz, 2H), 3.97 (t, J = 7.0 Hz, 2H), 1.78 (m, 2H), 1.44(m, 2H),1.36(m, 4H), 1.34(s, 12H), 0.92 (t, J = 7.0Hz, 3H) ppm.

2-(4-(dodecyloxy)phenyl)-4,4,5,5-tetramethyl-1,3,2-dioxaborolane(12b): Purge the high-pressure bottle by nitrogen for about 1 minute, then put $Pd(PPh_3)_2Cl_2$ (30 mg) in the high-pressure bottle, and then mix **12a** (0.50 g, 2.5 mmol) and the mixed solvent Et_3N/1,4-dioxane = 1/3 (v/v) of 12 mL into it and stir evenly with a magnet. Finally, add pinacoborane

(630 μL, 3.75 mmol) and bubble with nitrogen for about 2 minutes. After refluxing for 1 day, the mixture was cooled down to room temperature. Extracted twice with CH_2Cl_2/H_2O, and the organic layer was dried over anhydrous magnesium sulfate ($MgSO_4$). Purify via silica column chromatography (the eluent was n-hexane) to obtain transparent liquid. The yield is about 70%. ^{1}HNMR (400 Hz, $CDCl_3$): δ= 7.73 (d, 18 J = 9.2 Hz, 2H), 6.88 (d, J = 9.2 Hz, 2H), 3.97 (t, J = 7.0 Hz, 2H), 1.77 (m, 2H), 1.43(m, 2H), 1.34(s, 12H), 1.26(m, 16H), 0.88(t, J = 7.0Hz, 3H) ppm.

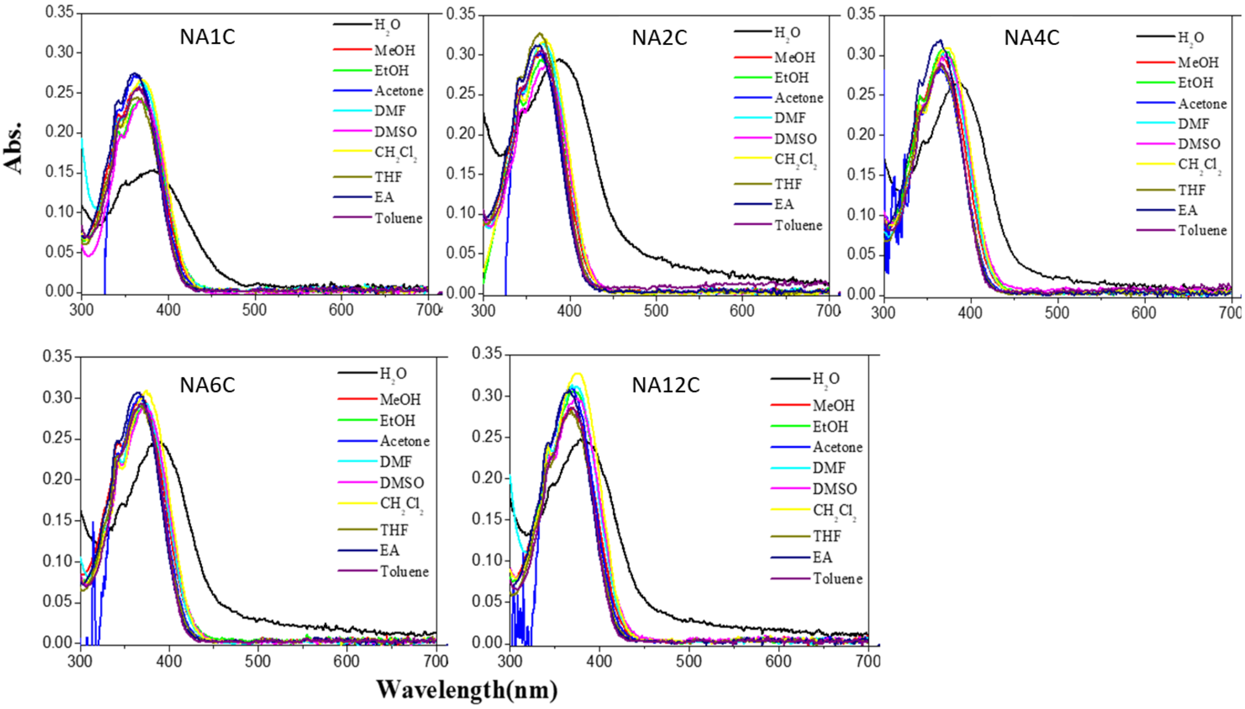

Figure S1: shows the absorption spectrum of NA1C, NA2C, NA4C, NA6C, and NA12C (20 μM) in different solvents (H_2O, MeOH, EtOH, Acetone, DMF, DMSO, CH_2Cl_2, THF, EA, and Toluene)

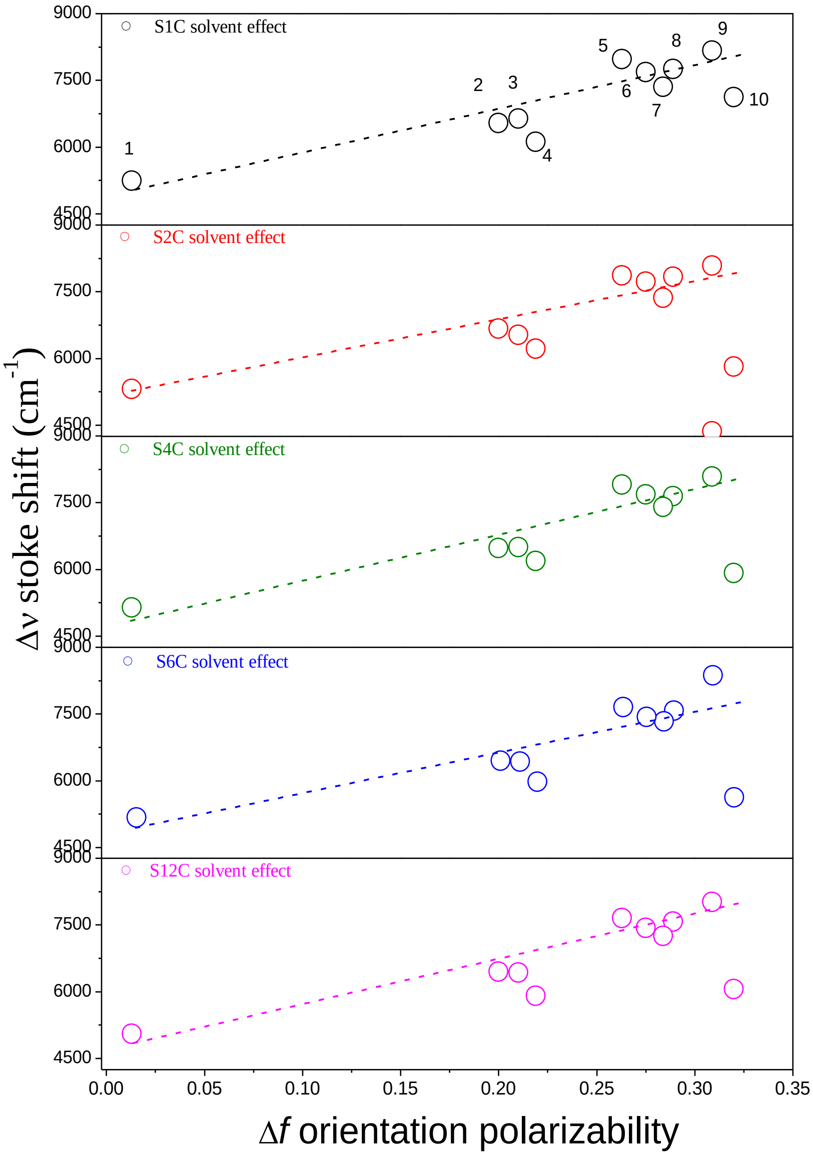

Figure S2: Lippert plots of compounds NAXC by solvent effect according to the solvent polarity parameter (1: Toluene, 2: EA, 3: THF, 4:CH_2Cl_2, 5: DMSO, 6: DMF, 7: Acetone, 8: EtOH,9: MeOH, 10: H_2O)

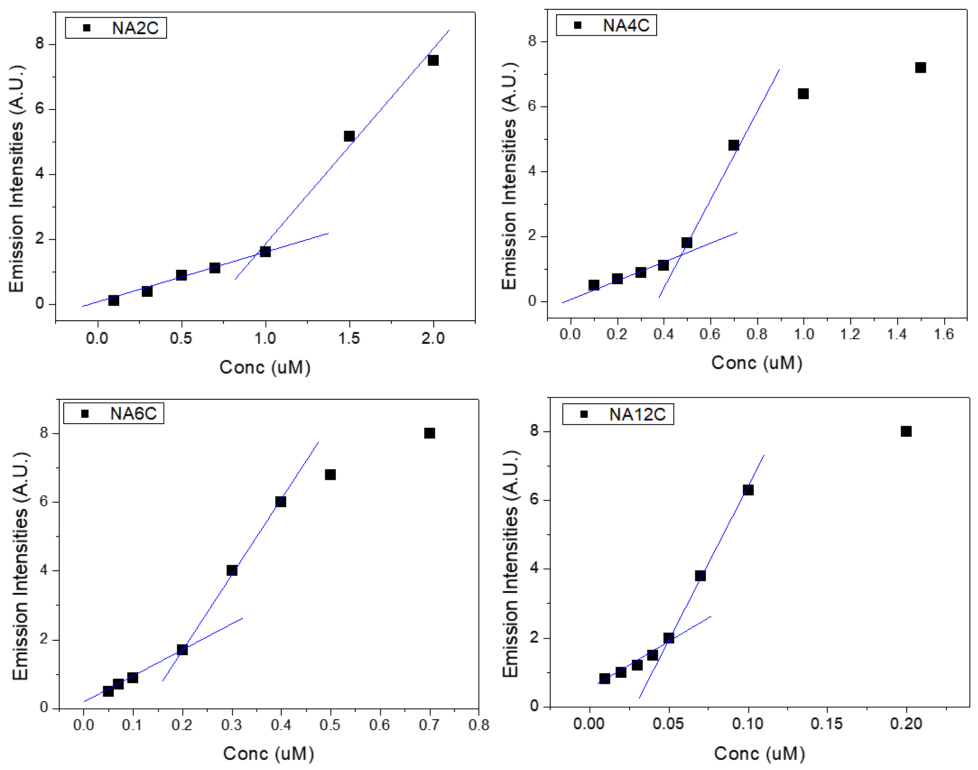

Figure S3. shows the critical microcapsule concentrations of compounds NA2C, NA4C, NA6C, and NA12C in H_2O.

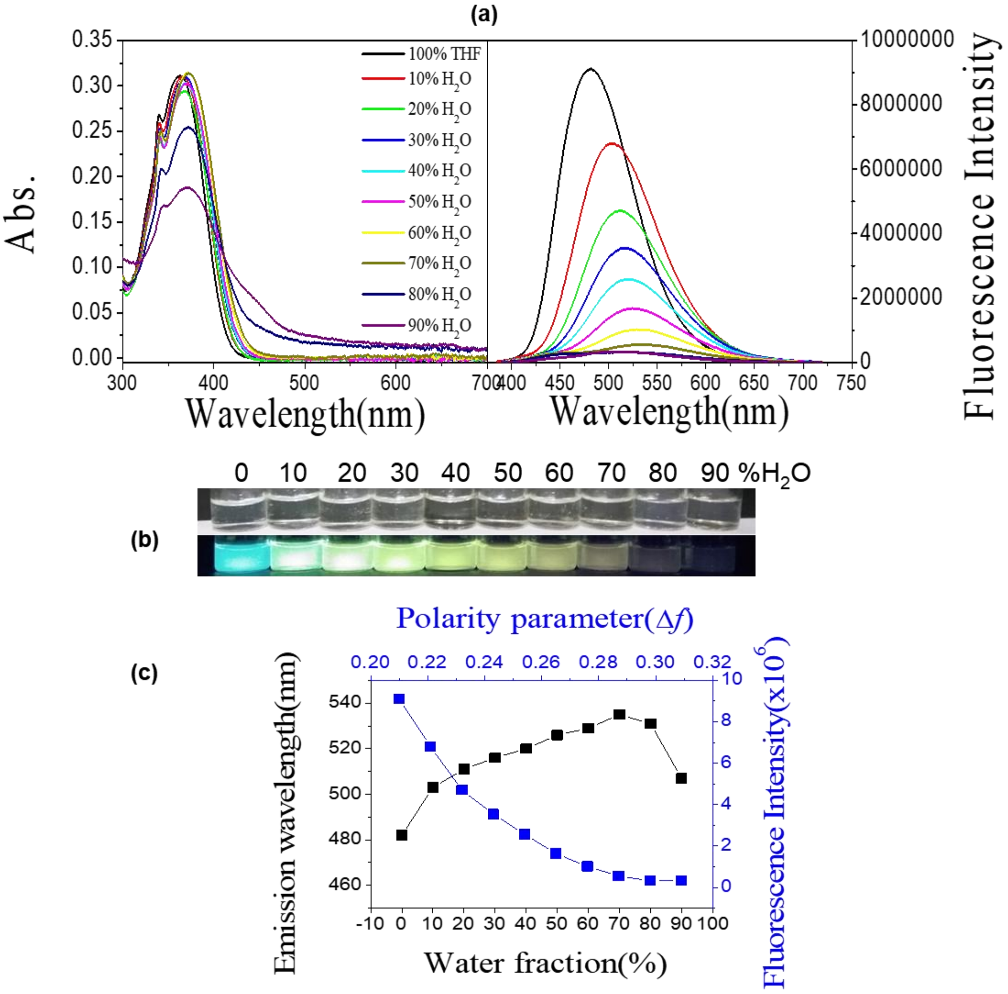

Figure S4. shows the (a) absorption and fluorescence spectra of 20μM NA1C a mixture solvent THF/H_2O. (b) images show the color change of the solutions under UV lamb (light source wavelength = 365nm). (c) Plot showing the relation between the water fractions, emission wavelengths and intensities, and polarities from (a).

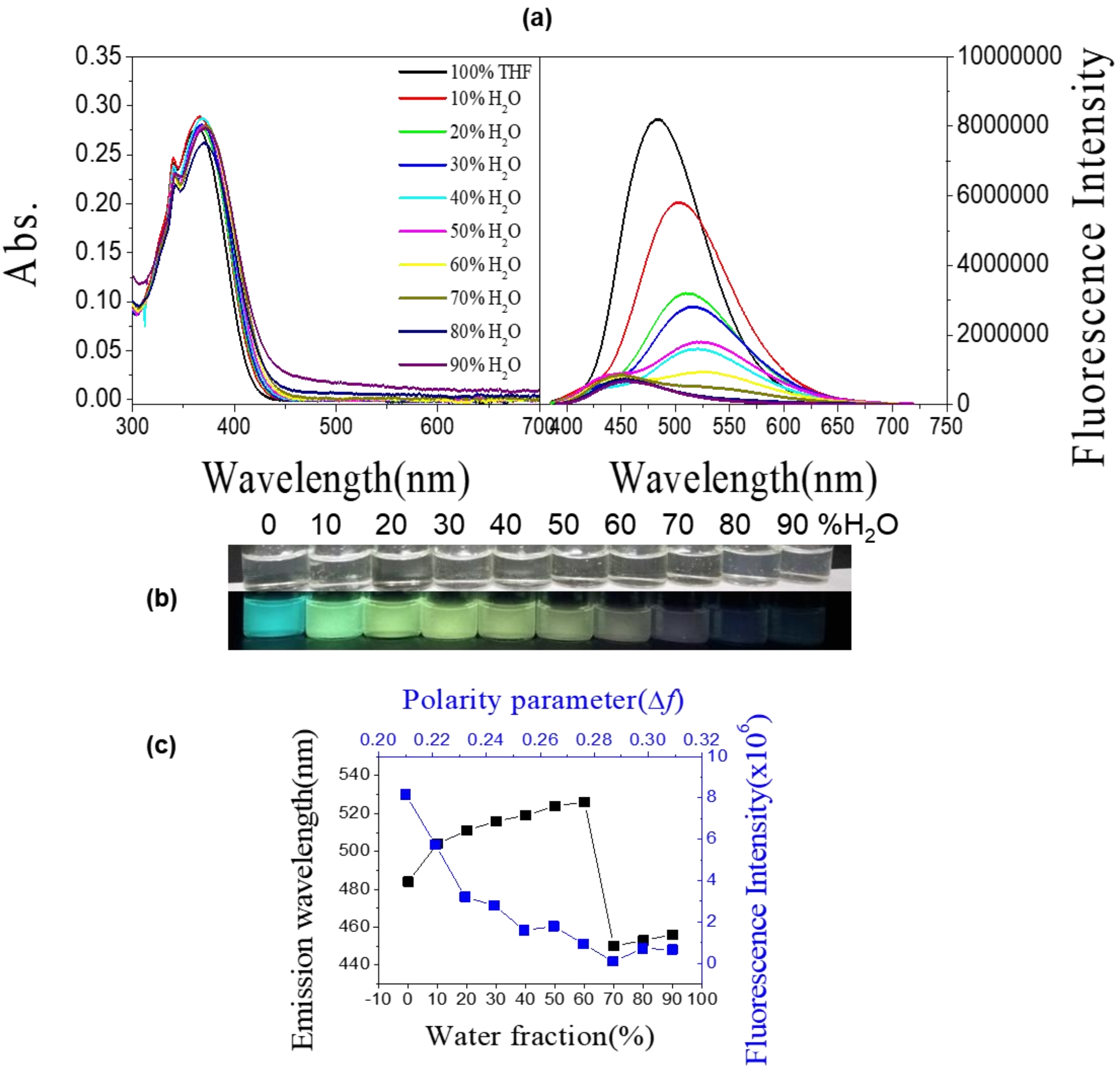

Figure S5. shows the (a) absorption and fluorescence spectra of 20μM NA2C a mixture solvent THF/H_2O. (b) images show the color change of the solutions under UV lamb (light source wavelength = 365nm). (c) Plot showing the relation between the water fractions, emission wavelengths and intensities, and polarities from (a).

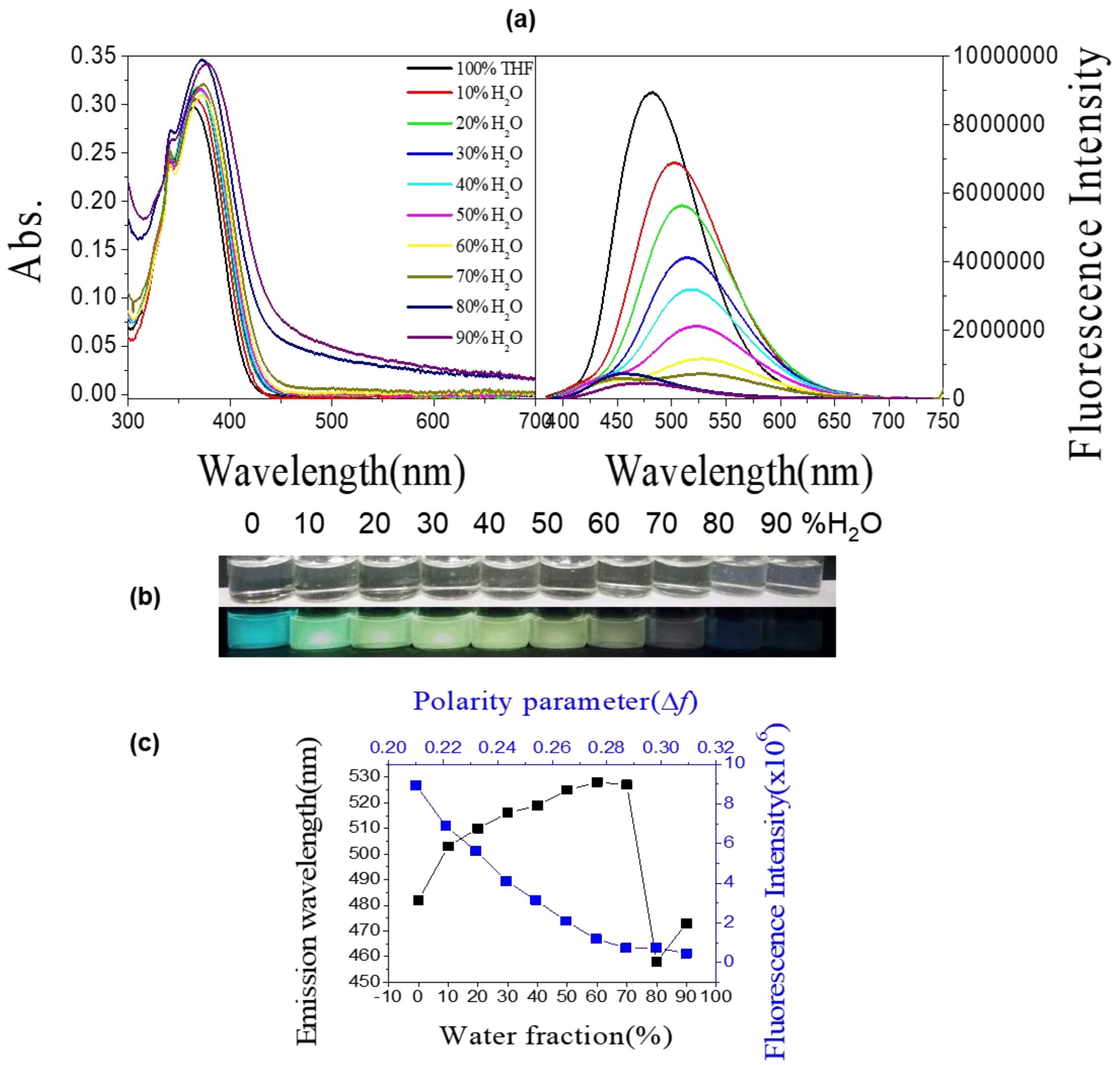

Figure S6. shows the (a) absorption and fluorescence spectra of 20μM NA4C a mixture solvent THF/H_2O. (b) images show the color change of the solutions under UV lamb (light source wavelength = 365nm). (c) Plot showing the relation between the water fractions, emission wavelengths and intensities, and polarities from (a).

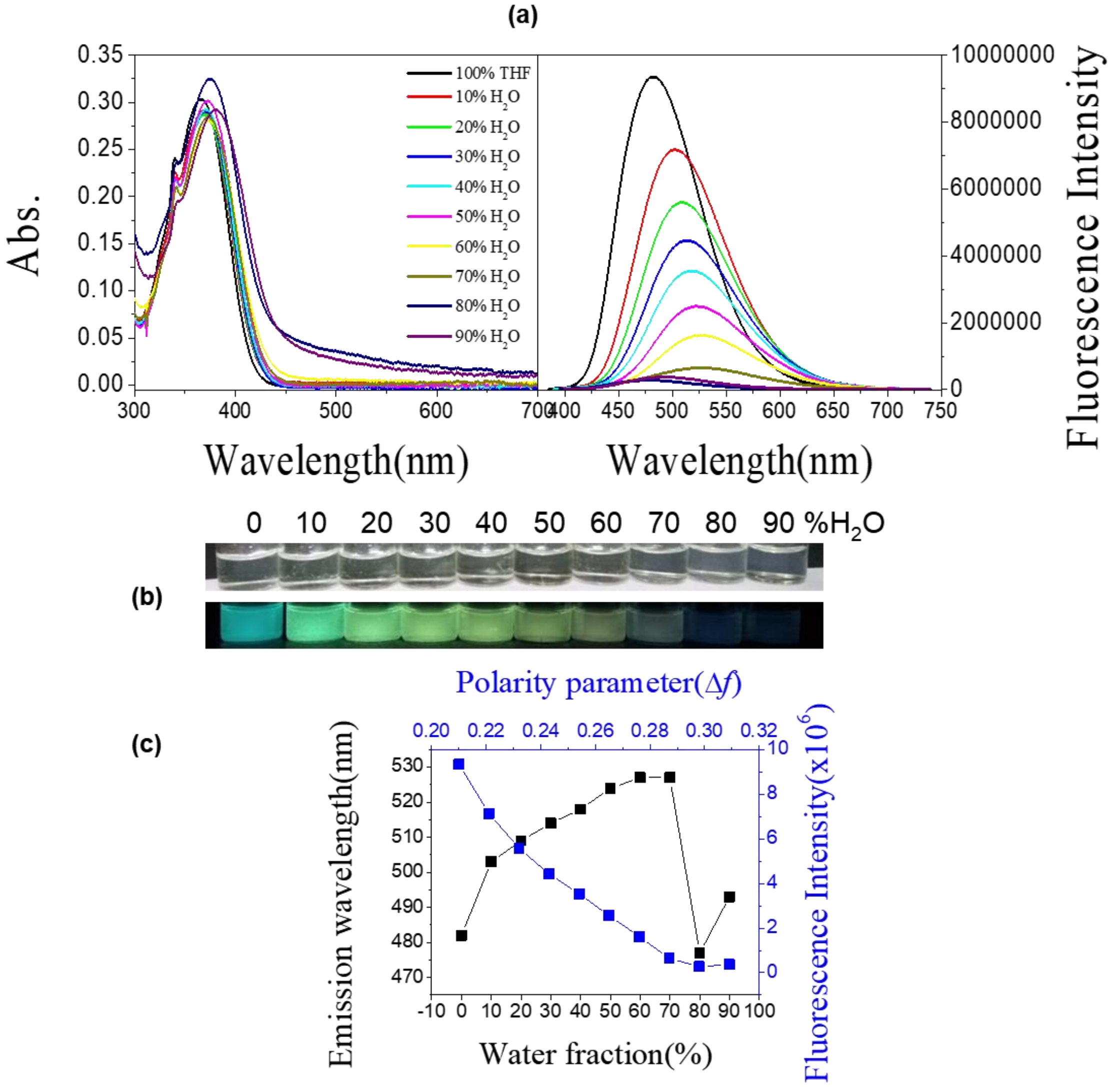

Figure S7. shows the (a) absorption and fluorescence spectra of 20μM NA6C a mixture solvent THF/H_2O. (b) images show the color change of the solutions under UV lamb (light source wavelength = 365nm). (c) Plot showing the relation between the water fractions, emission wavelengths and intensities, and polarities from (a).

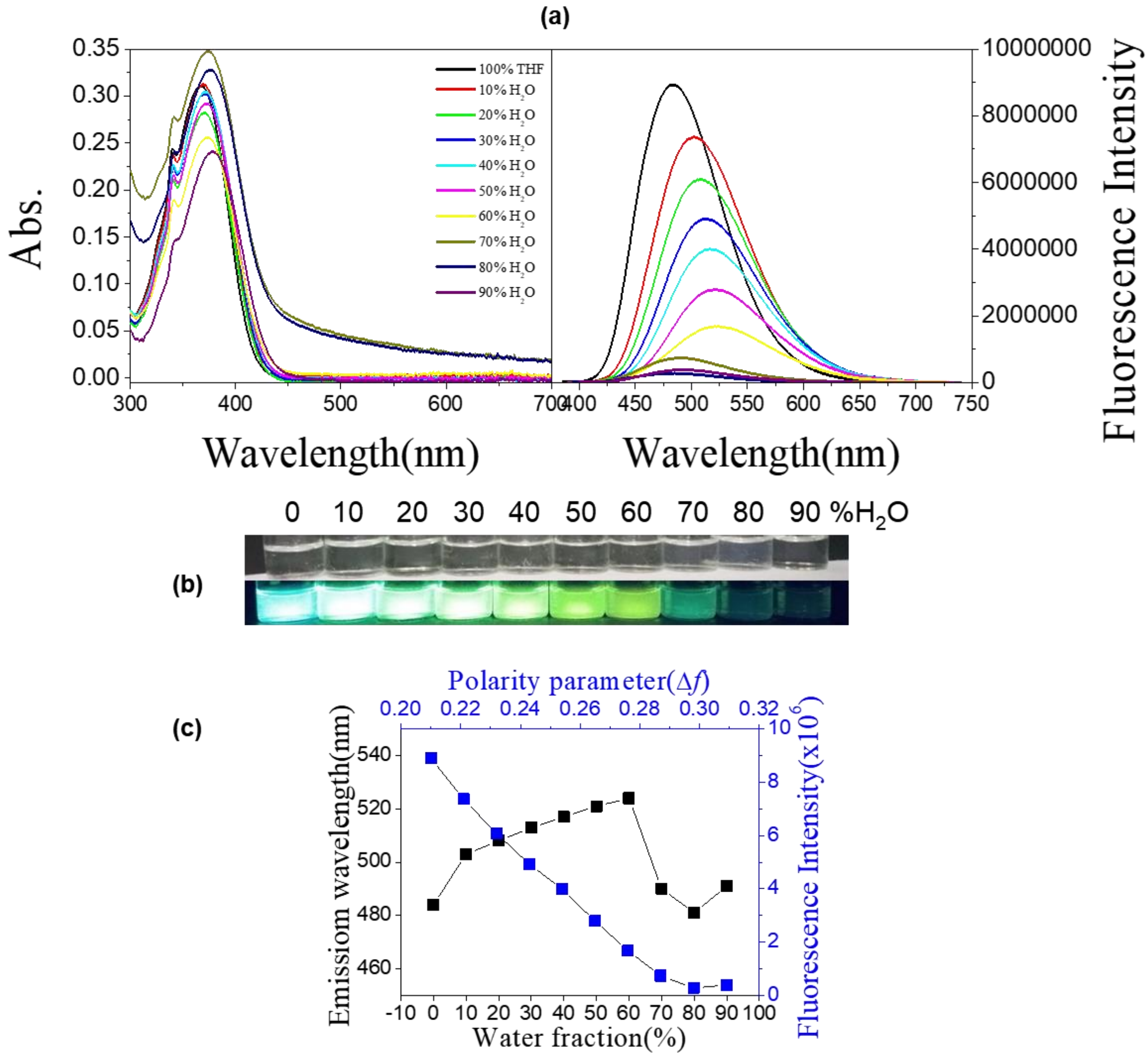

Figure S8. shows the (a) absorption and fluorescence spectra of 20μM NA12C a mixture solvent THF/H_2O. (b) images show the color change of the solutions under UV lamb (light source wavelength = 365nm). (c) Plot showing the relation between the water fractions, emission wavelengths and intensities, and polarities from (a).

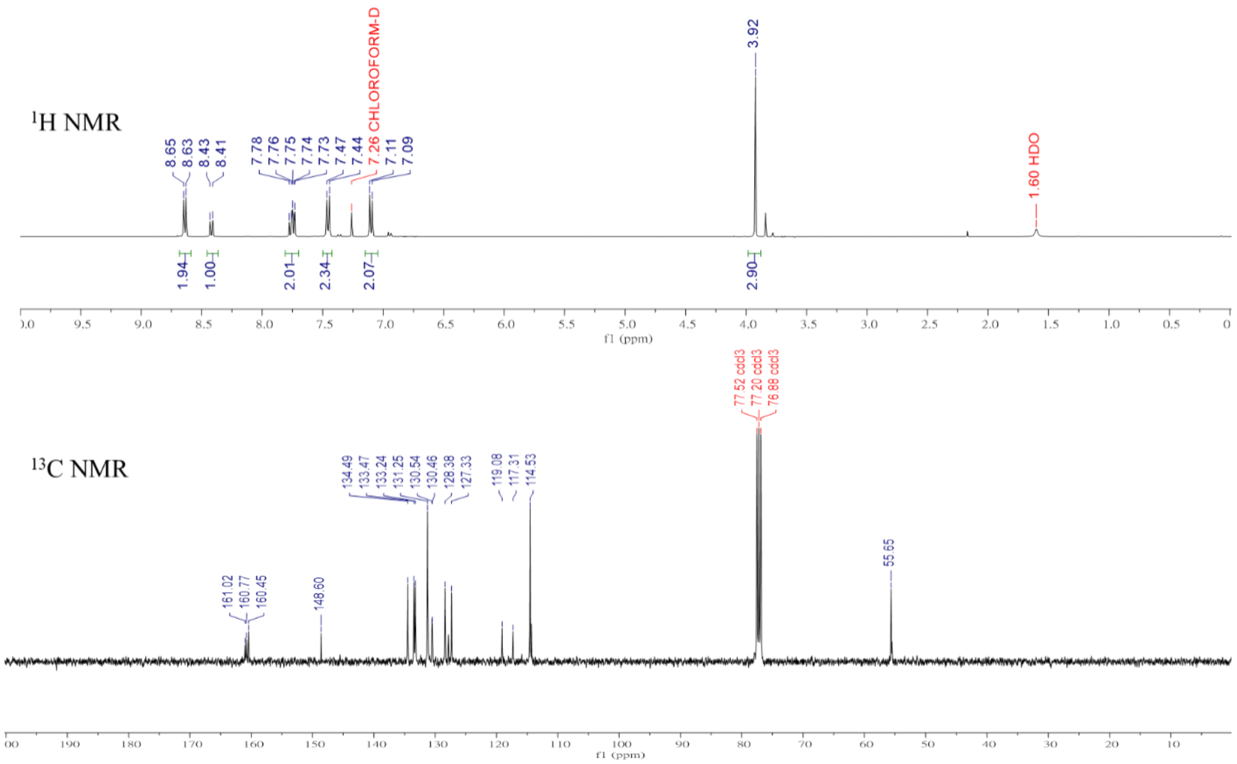

Figure S9. 400 MHz ^{1}H NMR spectrum (up) and ^{13}C NMR spectrum (down) in $CDCl_3$ of NA1C.

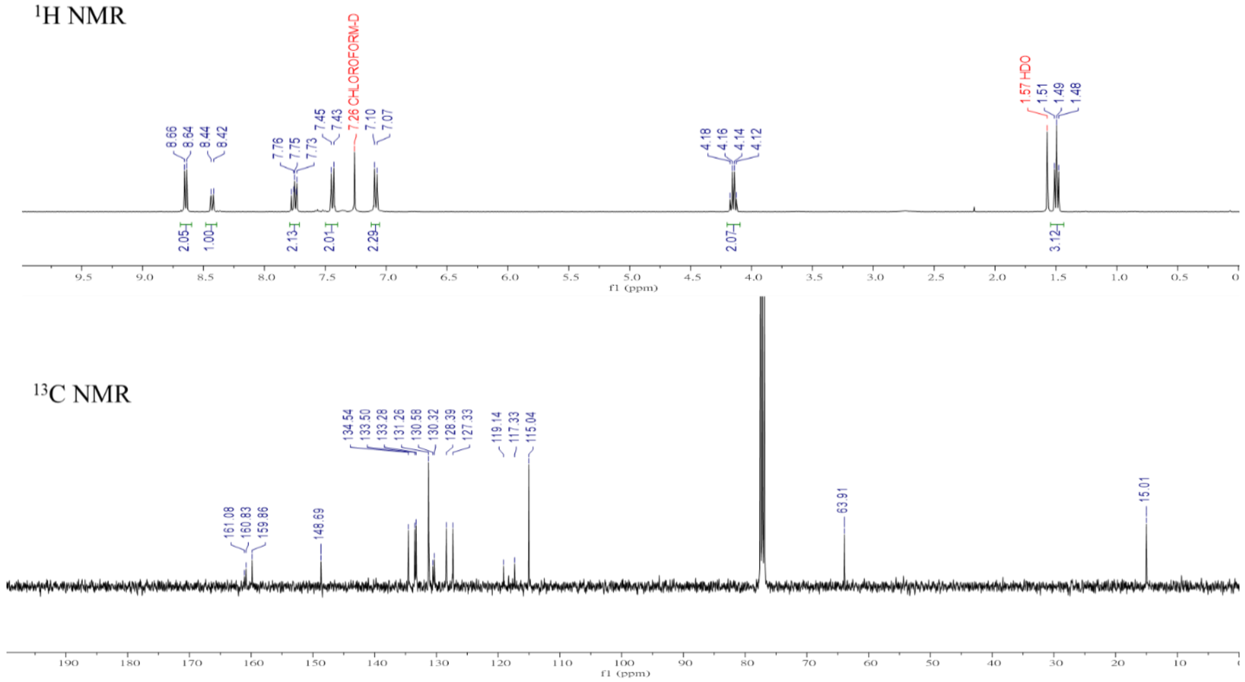

Figure S10. 400 MHz ^{1}H NMR spectrum (up) and ^{13}C NMR spectrum (down) in $CDCl_3$ of NA2C.

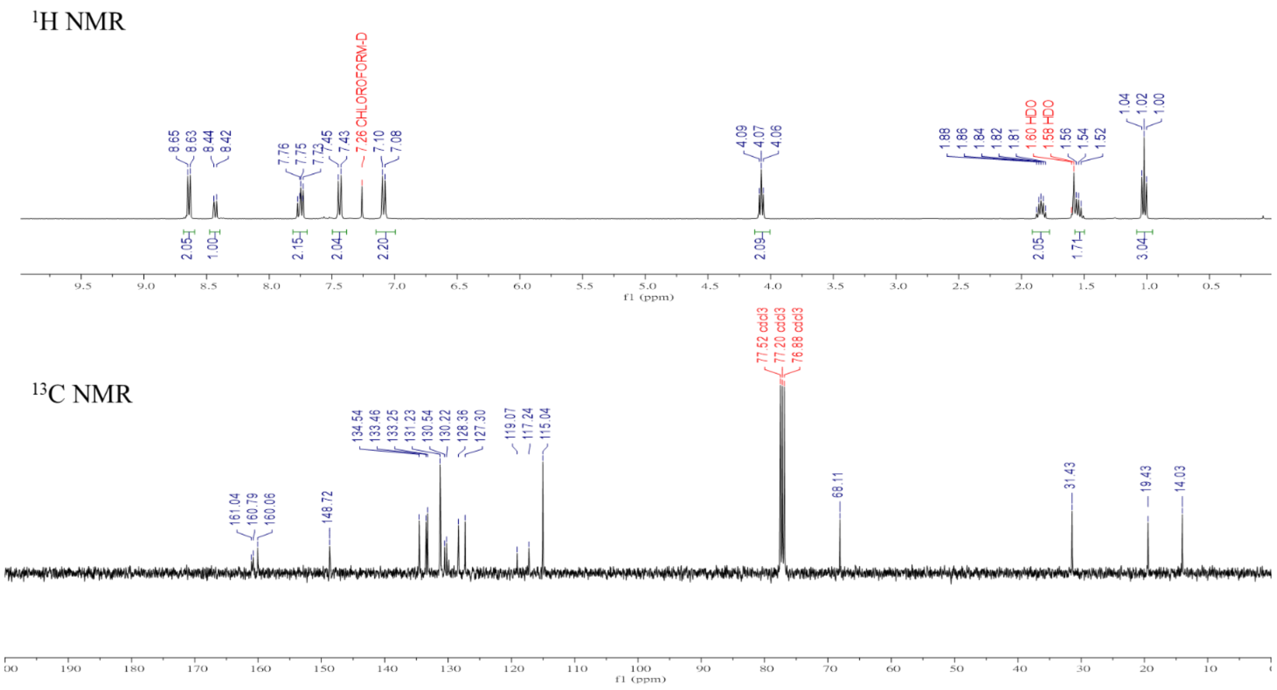

Figure S11. 400 MHz ^{1}H NMR spectrum (up) and ^{13}C NMR spectrum (down) in $CDCl_3$ of NA4C.

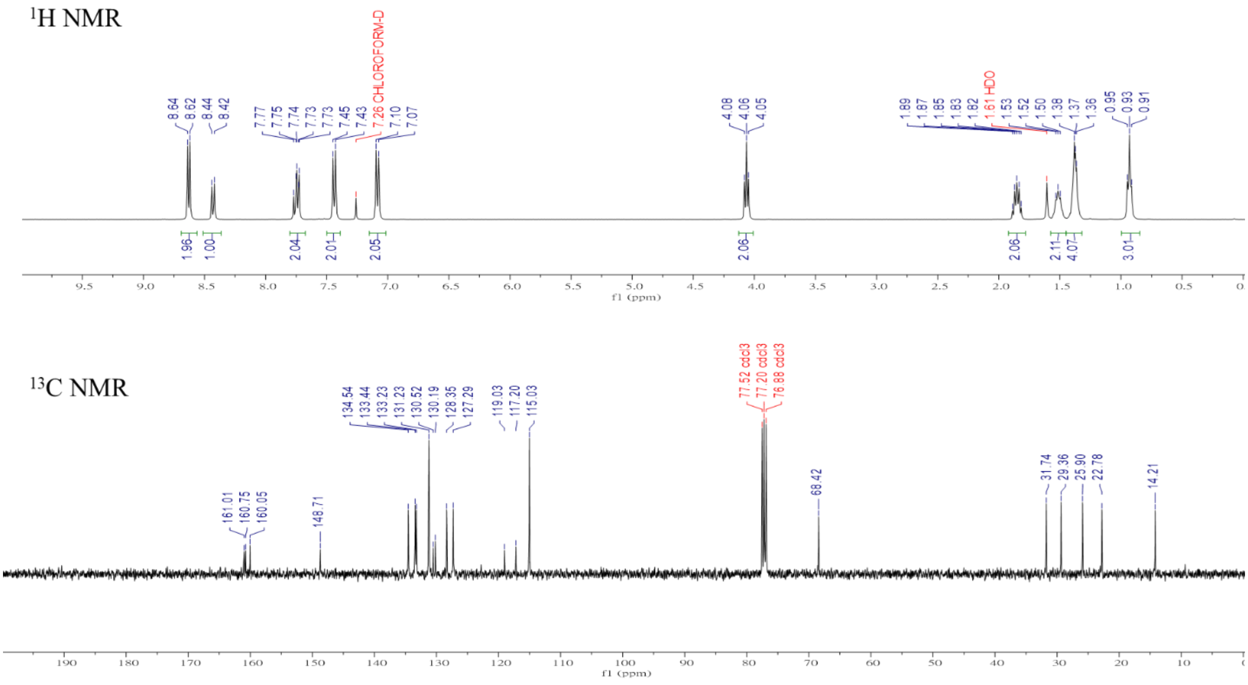

Figure S12. 400 MHz ^{1}H NMR spectrum (up) and ^{13}C NMR spectrum (down) in $CDCl_3$ of NA6C.

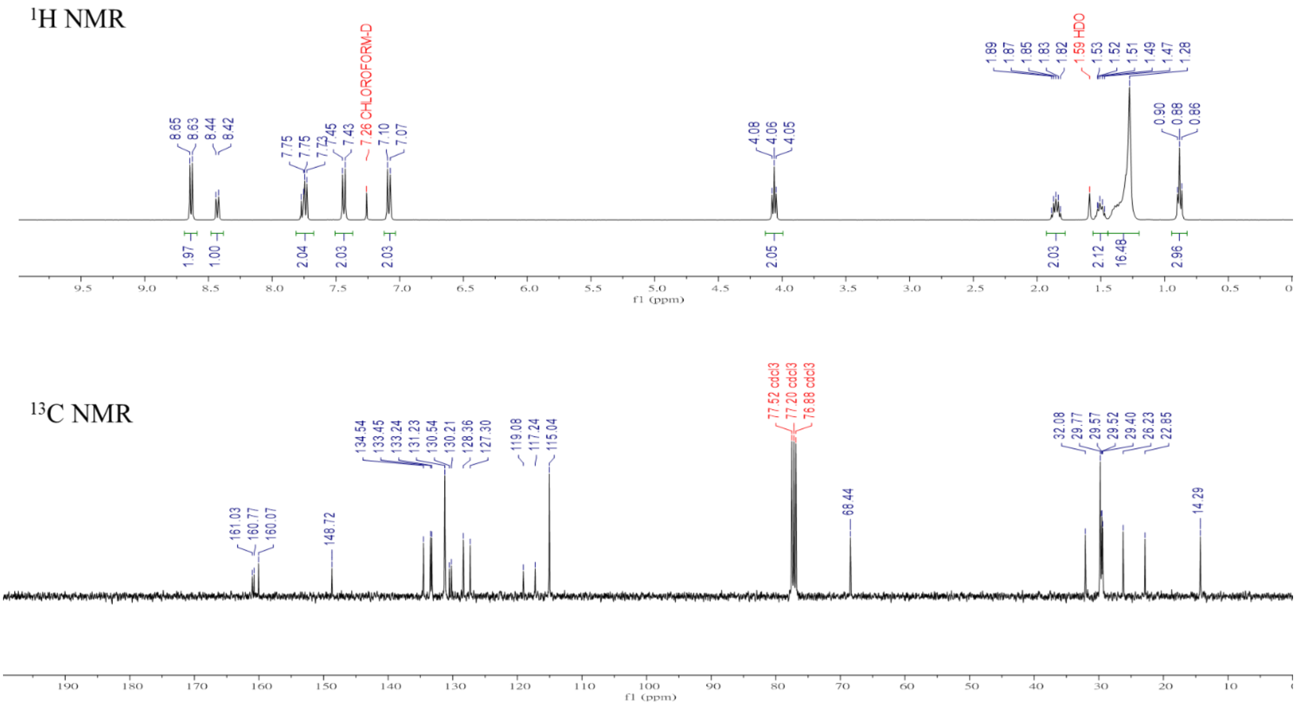

Figure S13. 400 MHz ^{1}H NMR spectrum (up) and ^{13}C NMR spectrum (down) in $CDCl_3$ of NA12C.

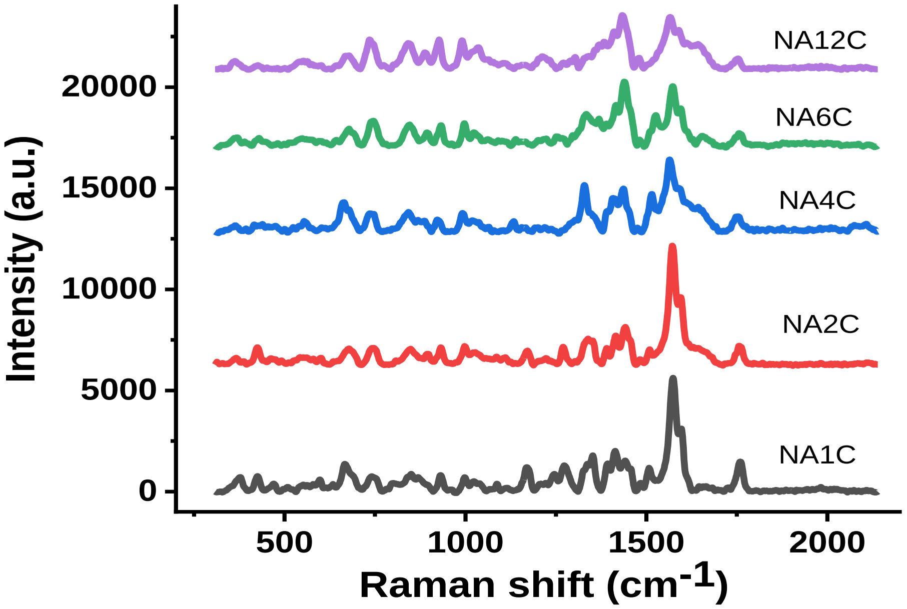

Figure S14. The Raman spectra of NAXC. Excited wavelength: 633 nm laser (poser: 0.35 mW), exposure time: 1s, accumulation number: 20 time
